# Editorial: Nutritional physiology of *Aquacultured species*

**DOI:** 10.3389/fphys.2022.1130143

**Published:** 2023-01-12

**Authors:** Mohamed Salah Azaza, Helena Peres, Serhat Turkmen

**Affiliations:** ^1^ Aquaculture Laboratory, National Institute of Marine Sciences and Technology, University of Carthage, Salammbô, Tunisia; ^2^ Department of Biology, Faculty of Sciences of University of Porto, Porto, Portugal; ^3^ Interdisciplinary Center for Marine and Environmental Research, Matosinhos, Portugal; ^4^ University of Alabama at Birmingham, Birmingham, AL, United States

**Keywords:** nutrient efficiency, digestibility, oxidative stress, digestive enzymes activities, feed management, metabolic response, feed additives

Whatever the aqua-cultured species, it is crucial that aqua feed be adequate and sustainable, as feeds represent the main contribution to production costs. In the face of this challenge, in the last few decades, substantial efforts have been oriented to identify alternatives to high-cost ingredients (e.g., fishmeal and fish oil) from unconventional protein sources and carbohydrates, particularly of vegetal origin, with variable success. These attempts may not be enough if the diet profile is not improved so as to boost and maintain the good health, welfare, and immune capacity of rearing species, abreast of growth performance, and feed conversion efficiency (Azaza et al., 2020). Therefore, the Research Topic “Nutritional Physiology of *Aquacultured species*” has been conceived to set out such knowledge and improve understanding of the relationships between nutrition and the related physiological aspects. This Research Topic comprises eleven original research articles.

The limited supply of fishmeal and fish oil associated with the continuous increase of their price has severely impacted farming costs. The search for feasible alternatives to fishmeal and fish oil is a prerequisite for sustainable aquaculture development. Significant progress has been made, and modern aquaculture diets are shifting towards agriculture-based ingredients. In this context, Biasato et al. investigated the growth performance, whole-body proximate composition, and intestinal microbiome of rainbow trout strains when selected and non-selected for weight gain on full-plant-based diets. Results demonstrate that the selected strain showed higher survival, final body weight, weight gain, and specific growth rate when compared to the non-selected strain. Furthermore, decreased whole-body lipid content was identified in the plant protein-fed selected rainbow trout compared to the non-selected strain fed the same diet.

Another study by Liu et al. was conducted on largemouth bass to investigate the effects of low, medium, and high viscous guar gums on growth performance, apparent nutrient digestibility, intestinal development, and morphology. Results indicated that guar gum diets adversely affected intestinal morphology, decreased intestinal digestive and absorptive enzyme activities, and caused poor nutrient digestibility and growth performance in juvenile largemouth bass. In fact, the adverse effects of guar gum are closely related to its viscous level, and high viscous guar gum adversely affects the rearing performance of juvenile largemouth bass.

In addition to the above-mentioned fish species, two investigations were conducted on Pacific white leg shrimp (*Litopenaeus vannamei*)*,* considered the most important farmed crustacean species. Peng et al. evaluated the effects of increased dietary inclusion of soybean meal on growth performance, apparent digestibility, intestinal digestive enzyme activity, and muscle growth-related gene expression. Results indicated that the final body weight, weight gain, specific growth rate, feed intake, intestinesomatic index, and dressed weight percentage linearly and significantly decreased as dietary soybean meal increased from 20% to 50%. The same trend was also observed in the apparent digestibility coefficients of dry matter, crude protein, crude lipid, and ash. For the same species (i.e., *Litopenaeus vannamei*), Hi et al. investigated the effects of replacing commercial feeds with fresh black soldier fly larvae (BSFL) on the intestinal microbiota, immune enzyme activities, and rearing water quality. Authors reported that proper replacement of commercial feed with fresh BSFL had positively modulated intestinal health and immune-related enzyme activities, as well as the water quality.

Increased diversity of non-nutritive or functional aquafeed additives has been used as a valuable approach to stabilize feed quality, enhance growth, digestibility, feed efficiency, and immune status, and as an alternative strategy for disease-fighting. Functional feed additives include phytogenic compounds, mycotoxin binders, organic acids, immunostimulants, yeast products, probiotics, prebiotics, and enzymes. In this context, four research articles featured in the current Research Topic dealt with using feed additives. Peng et al. investigated the effects of dietary condensed tannins, regarded as a potent antioxidant, anti-inflammatory, and antibacterial activities, on serum metabolites, antioxidant and immune response, liver histomorphology, and glycometabolism enzyme activities of *Chinese seabass (Lateolabtax maculatus)*. Results revealed that condensed tannins dietary supplementation, up to 2 g/kg of diet, reduced serum lipid and glucose levels, enhanced liver antioxidant and immune response, and improved glucose utilization of *L. maculatus*.

The study conducted by Mansour et al. aimed to investigate the effects of increasing dietary supplementation levels with an ethanolic extract from *A. platensis* naturally rich in astaxanthins (circa 98%) on growth performance, feed utilization, immune-related genes expression, and water and intestinal microbiota of Pacific white leg shrimp*.* Supplementing a commercial diet with 4 g kg^−1^ crude *A. platensis* extract did not affect the survival rate and significantly improved shrimp growth performance and feed conversion ratio compared to the control diet. The superoxide dismutase and immune-related gene expression (*prophenoloxidase*, *lysozyme*, *beta-glucan binding protein*, *transglutaminase*, and *crustin*) were significantly upregulated in groups fed increasing levels of this extract. Besides, results demonstrate that increasing *A. platensis* extract supplementation levels significantly reduced the prevalence of heterotrophic bacteria and *Vibrio* spp*.*

Wang et al. investigated the effects of glutathione (GSH), as a feed additive in practical diets, on growth, intestinal antioxidant capacity, intestine histology, gene expression, and gut microbiota in juvenile triploid rainbow trout (*Oncorhynchus mykiss*). Based on the broken-line regression analysis, results demonstrate that the optimum dietary GSH level to maximize growth performance was circa 447 mg kg^−1^ of diet. Likewise, feeding juvenile triploid *O. mykiss* 200–800 mg kg^−1^ GSH increased intestinal catalase and superoxide dismutase activities and improved general intestinal health.

Another study on dietary supplementation with a commercial feed additive, betaine, a by-product of sugar beet processing widely used as an attractant, was conducted by Li et al. This study aimed to investigate the mechanism by which betaine modulates reactive oxygen species (ROS) production *via* Wnt10b/β-catenin signaling in zebrafish liver, based on the fact that, under oxidative stress, chronically elevated ROS levels play a crucial role in innate fish immunity. Results showed that betaine enrichment of diet at levels of .1, .2, and .4 g/kg induced Wnt10b and β-catenin gene expression but suppressed GSK-3β expression in zebrafish liver. In addition, irrespective of the betaine supplementation level, betaine led to a reduction of superoxide anion (O_2_^·−^), hydrogen peroxide (H_2_O_2_), and hydroxyl radical (·OH) content. However, dietary betaine enrichment, at .1, .2, or .4 g/kg diet, upregulated hepatic gene expression of antioxidant enzymes and increased activity of superoxide dismutase (SOD), glutathione peroxidase (GSH-PX) and catalase (CAT) in zebrafish, clearly demonstrating that betaine can efficiently inhibit ROS production.

Understanding the functionality of the digestive tract is a prerequisite to optimizing diet formulation for new species. Knowledge of fish digestive biochemistry and its health is essential in determining animal performance, feed utilization efficiency, and aquaculture profitability. To strengthen understanding on lumpfish (*Cyclopterus lumpus*) nutrition, a rapidly expanded cultured species in the last decade, Zhou et al. evaluated the effects of various dietary macronutrient compositions on gut function. The results demonstrated that increased lipid and decreased protein levels in the diet negatively impacted digestive function, including the reduced activity of brush border membrane digestive enzymes and gene expression related to nutrient digestion and transport, ion exchange, immune regulation, and cell remodeling. The effect of dietary lipid to carbohydrate ratio (7.5/18.3, 13.8/14.6, and 18.1/9.5) of isoproteic diets (55% crude protein) on macronutrient digestibility was also studied. Results showed that decreased as the starch level increased, whereas protein digestibility was not affected by the lipid/carbohydrate ratio. This led to conclude that protein sparing effect of lipids negatively affected digestion, absorption, and immune responses in the lumpfish intestine.

Greater amberjack (*Seriola dumerili*) is a pelagic teleost highly interested in marine aquaculture diversification, high growth rates, and exquisite flesh quality. To further acquire scientific knowledge on the digestive physiology of this species, Navarro et al. studied the activity and functional characteristics of key digestive enzymes (i.e., pepsin, trypsin, chymotrypsin, etc.) and the modulatory effect of water temperature. Results demonstrate that chymotrypsin was the most active enzyme in the digestive tract of the greater amberjack, while lipase was the enzyme with lower activity. The activity of trypsin, chymotrypsin, and lipase was responsive to water temperature, even though the highest activity of trypsin was reached at 26°C and of chymotrypsin at 18°C.

The last paper concerns the pearl oysters, *Pinctada fucata martensii* and *P. maxima*, which are the two main farmed species used for producing nucleated round pearls. Ye et al. evaluated the growth performance, physiological energetics, and activity of digestive enzymes and carbonic anhydrase of both species fed with different microalga diets. Results showed that the relative growth rate (RGR) of *P. f. martensii* was higher than that of *P. maxima*. Irrespective of the microalga species, RGR was higher when fed with a microalgae bend than with a single microalga. Amylase, cellulase, lipase, and pepsin activity was higher in *P. f. Martensii* than with *P. maxima* fed with the same diets.

The papers presented in this Research Topic and conducted on diverse species highlight the usefulness of the physiologic approaches to deepen knowledge in feeding farmed aquatic organisms. This helps fish nutritionists to tailor and improve the nutritional profile of the diet and hence to provide more adequate and healthy diets for fish. We sincerely thank all authors and reviewers for their valuable contribution that made the publication of this Research Topic possible.
